# Next-generation polymeric nanoparticles for regenerative endodontics: engineering the pulp microenvironment beyond antimicrobial therapy

**DOI:** 10.3389/fdmed.2026.1972546

**Published:** 2026-09-28

**Authors:** Rosana Farjaminejad, Neeki Jabbari, Alexander Garcia-Godoy, Franklin Garcia-Godoy, Elnaz Tavazozadeh

**Affiliations:** 1 Department of Health Services Research and Management, School of Health and Psychological Sciences, City, University of London, London, United Kingdom; 2 Independent Researcher, Toronto, Canada,; 3 Advanced Endodontic Solutions, Tamarac, FL, United States; 4 Department of Bioscience Research, Bioscience Research Center, College of Dentistry, University of Tennessee Health Science Center, Memphis, TN, United States; 5 Department of Orthodontics, Georgia School of Orthodontics, Atlanta, GA, United States

**Keywords:** angiogenesis, dental pulp regeneration, dental pulp stem cells, immunomodulation, odontogenic differentiation, polymeric nanoparticles, pulp microenvironment, regenerative endodontics

## Abstract

Regenerative endodontics aims not only to control infection but also to restore a functional pulp-dentin complex. Achieving this predictably remains challenging because disinfection, inflammation, blood vessel formation, matrix remodeling, and stem cell behavior are closely connected. Polymeric nanoparticles are increasingly being explored as a way to influence this complex environment because they can deliver therapeutic agents in a controlled and, in some cases, responsive manner. This review examines how the pulp microenvironment affects regenerative outcomes and discusses nanoscale strategies that target key aspects of this process, including infection-driven inflammation, macrophage behavior, hypoxia and vascular dysfunction, matrix reconstruction, and odontogenic differentiation. Current evidence suggests that nanoparticle performance depends not only on the cargo itself, but also on how it is released, the material design, responsiveness to biological signals, and the timing of delivery. These factors can significantly influence the behavior of regenerative cells. However, most studies remain preclinical, and no single platform has yet been able to coordinate all of these functions throughout the entire process of pulp regeneration. Future approaches will likely need to move beyond simply combining several therapeutic agents in one particle and instead focus on systems that can respond to changes in the pulp microenvironment over time. Whether these systems can do so while maintaining cytocompatibility, reproducibility, manufacturability, and clinical practicality will be important for their future use in regenerative endodontics.

## 1 Introduction

Conventional root canal treatment remains the standard therapy for managing pulpal infection by eliminating microorganisms and sealing the root canal system. Although this approach is highly successful in preserving the tooth, it does not restore the biological functions of the pulp, including immune defense, vascularization, sensory perception, and dentin deposition (1). Farjaminejad et al. emphasized that regenerative endodontic therapy (RET) represents a biological alternative aimed at restoring the pulp–dentin complex through a coordinated “tissue engineering triad” of stem cells, scaffolds, and signaling molecules, rather than simply replacing necrotic tissue with inert filling materials (2). However, even when RET achieves its primary clinical goal of resolving infection, histological evidence indicates that the newly formed tissue within the canal space is frequently periodontal- or osseous-like (cementum, bone, and periodontal-ligament-like tissue) rather than a true pulp–dentin complex. On this basis, RET has been characterized as a *reparative* rather than a *regenerative* process, with root maturation, apical closure, and return of neurogenesis all occurring unpredictably even under standardized protocols (3). This distinction between repair and true regeneration, together with patient-centered drawbacks such as crown discoloration and the need for multiple appointments, underscores that current RET strategies have not yet closed the gap between clinical success and biological restoration (3).

Recent advances in regenerative endodontics have shifted the treatment objective further toward biological regeneration, yet variability in clinical outcomes and the absence of standardized treatment protocols continue to limit predictable regeneration (4). A central obstacle is the disinfection–biocompatibility trade-off: sodium hypochlorite and triple antibiotic paste, while effective against intracanal biofilm, are cytotoxic to stem cells of the apical papilla and dental pulp stem cells (DPSCs) at concentrations conventionally used, compromising the very cell populations required for regeneration (5). In parallel, immunomodulation has emerged as an independent determinant of regenerative success, as macrophage polarization toward an anti-inflammatory (M2) phenotype can enhance tissue repair and reduce inflammation within the root canal microenvironment. This effect is distinct from, and not achieved by, antimicrobial action alone (5). These observations indicate that future biomaterials must do more than disinfect; they must actively regulate the local cellular and immune microenvironment.

Advances in biomaterial science have introduced bioactive materials, calcium silicate-based cements, nanofibrous scaffolds, and injectable hydrogels that provide not only structural support but also cues for cell adhesion, differentiation, and mineralization (6). Notably, nanoparticles such as silver, gold, and bioactive glass nanoparticles are increasingly being incorporated directly into endodontic scaffolds, where they confer antimicrobial activity and promote mineralization simultaneously, illustrating that nanotechnology is already being explored as a multifunctional adjunct rather than a purely antimicrobial tool (2). Building on this, nanotechnology has emerged as a strategy to overcome several biological limitations of RET. Functionalized nanoparticles enable localized and temporally controlled delivery of bioactive molecules, improving stem-cell viability and regulating differentiation while creating a more favorable microenvironment for pulp–dentin regeneration (7). The development of smart nanoparticles capable of responding to environmental stimuli such as pH, temperature, and reactive oxygen species has further expanded the therapeutic potential of nanomaterials beyond passive drug delivery toward biologically responsive systems capable of tailoring their activity to the inflamed or infected pulp microenvironment (8).

Complementing these material-based strategies, recent experimental evidence demonstrates that successful pulp-dentin regeneration depends on recreating the developmental microenvironment of the pulp rather than delivering a single therapeutic agent. Odontogenic exosomes derived from DPSCs undergoing early odontogenic differentiation, acting through the transfer of microRNAs and other bioactive cargo, markedly enhanced odontogenesis, angiogenesis, and neurogenesis and achieved complete pulp–dentin complex regeneration *in vivo*, in contrast to exosomes from undifferentiated cells (9). This finding reinforces the principle that regeneration requires coordinated, multi-signal biological instruction, and it suggests that polymeric nanoparticles engineered as carriers for such cargo (miRNAs, growth factors, or exosomes) could extend this developmental-mimicry strategy into a more clinically translatable, cell-free platform (9).

Despite this scientific momentum, clinical adoption of regenerative and biomaterial-based endodontic strategies remains inconsistent; a multinational survey of specialists identified inadequate training, uncertainty regarding outcomes, and limited clinical experience as principal barriers to broader implementation (10). Collectively, these advances suggest that the next generation of regenerative endodontic therapies should move beyond simple antimicrobial strategies toward materials capable of actively regulating the pulp microenvironment: disinfecting without cytotoxicity, modulating the immune response, delivering bioactive signaling cargo, and supporting stem-cell-driven tissue formation. Among the available biomaterials, polymeric nanoparticles are uniquely positioned to integrate controlled drug delivery, immunomodulation, stem-cell support, and stimuli-responsive behavior, providing the foundation for more predictable and biologically driven regenerative outcomes.

## 2 Methods

This narrative review was developed through a literature search of PubMed, Scopus, Web of Science, and ScienceDirect, supplemented by Google Scholar to identify additional relevant studies. ResearchGate was used only to locate full texts or author-shared versions of potentially relevant articles, not as a primary bibliographic database. The search included studies published up to August 2026, with no predefined lower date limit, and was last updated in August 2026. Search terms were combined around regenerative endodontics, dental pulp regeneration, polymeric nanoparticles, nanocarriers, stimuli-responsive systems, dental pulp stem cells (DPSCs), stem cells from the apical papilla (SCAP), inflammation, macrophage polarization, hypoxia, angiogenesis, extracellular matrix (ECM), and odontogenic differentiation. Boolean combinations of these terms were used across databases, and reference lists of relevant articles were hand-searched to identify additional relevant studies.

English-language original studies and relevant reviews were considered, with particular emphasis on recent experimental evidence and studies addressing mechanisms directly related to regulation of the pulp microenvironment. Studies were included when they examined polymeric nanoparticles or nanoscale delivery systems relevant to infection control, immune regulation, vascularization, matrix reconstruction, or stem-cell behavior in regenerative endodontics. Because direct evidence for polymeric nanoparticles in this setting remains limited, selected non-endodontic or non-polymeric systems, including nanocomposite hydrogels, extracellular vesicles, inorganic nanomaterials, and other responsive nanoscale platforms, were also considered when they demonstrated a mechanism or design principle directly relevant to these functions. These included stimuli-responsive release, immunomodulation, angiogenic regulation, ECM-associated signaling, or sequential delivery. These studies were treated as supporting or conceptual evidence rather than direct evidence for endodontic polymeric nanoparticles, and their experimental context, model system, and limitations are identified where they are discussed. Accordingly, direct polymeric nanoparticle studies in dental or regenerative endodontic models are treated as direct evidence, whereas non-polymeric, non-endodontic, and related nanoscale systems are used as supporting mechanistic or engineering evidence. Studies without a clear mechanistic or engineering connection to pulp-microenvironment regulation were not considered.

The selected literature was synthesized according to the major biological components of the pulp microenvironment: infection-associated inflammation, immune regulation, hypoxia and vascular dysfunction, extracellular-matrix remodeling, and stem-cell fate. These components were then linked to nanoparticle strategies capable of influencing each process.

## 3 Why the pulp microenvironment determines regeneration

The pulp–dentin complex does not regenerate in isolation; its regenerative outcome is shaped by a set of interlocking biological signals that can either converge to support tissue formation or compound one another to impair it. Infection triggers an inflammatory cascade that consumes local oxygen and disrupts microvascular integrity, producing a hypoxic niche. This hypoxia, in turn, contributes to shaping the balance between pro-inflammatory (M1-like) and reparative (M2-like) macrophage phenotypes, thereby influencing inflammatory and pro-angiogenic signaling (11, 12). Vascular recovery then governs oxygen and nutrient delivery to the ECM, shaping whether the matrix is degraded further or remodeled into a scaffold capable of guiding cell behavior. Because resident and recruited stem cells read their differentiation cues directly from this matrix and its surrounding cytokine milieu, the same microenvironmental cascade that begins with infection ultimately determines whether stem cells commit to odontoblast-like, osteogenic, or fibrous lineages (13). Regeneration therefore succeeds or fails not because of any single factor in isolation, but because inflammation, hypoxia, immune polarization, matrix state, and stem-cell fate interact through overlapping and reciprocal signaling networks.

### 3.1 Infection-induced inflammation

Because the pulp is enclosed within rigid, mineralized dentin walls, its capacity for lymphatic drainage is anatomically restricted. Bacterial antigens and metabolic by-products that diffuse through dentinal tubules therefore provoke an inflammatory response whose fluid and cellular mediators cannot escape efficiently: extravasated fluid accumulates, interstitial pressure rises, blood vessels are compressed, and intrapulpal blood stasis develops, predisposing the tissue to hypoxia and necrosis if the stimulus is not resolved quickly (Figure 1) (14). This structural confinement means that the same cascade that initiates pathogen clearance also carries an intrinsic risk of self-amplification within a non-compliant space, a vulnerability unique to the pulp relative to most other soft tissues.

**Figure 1:**
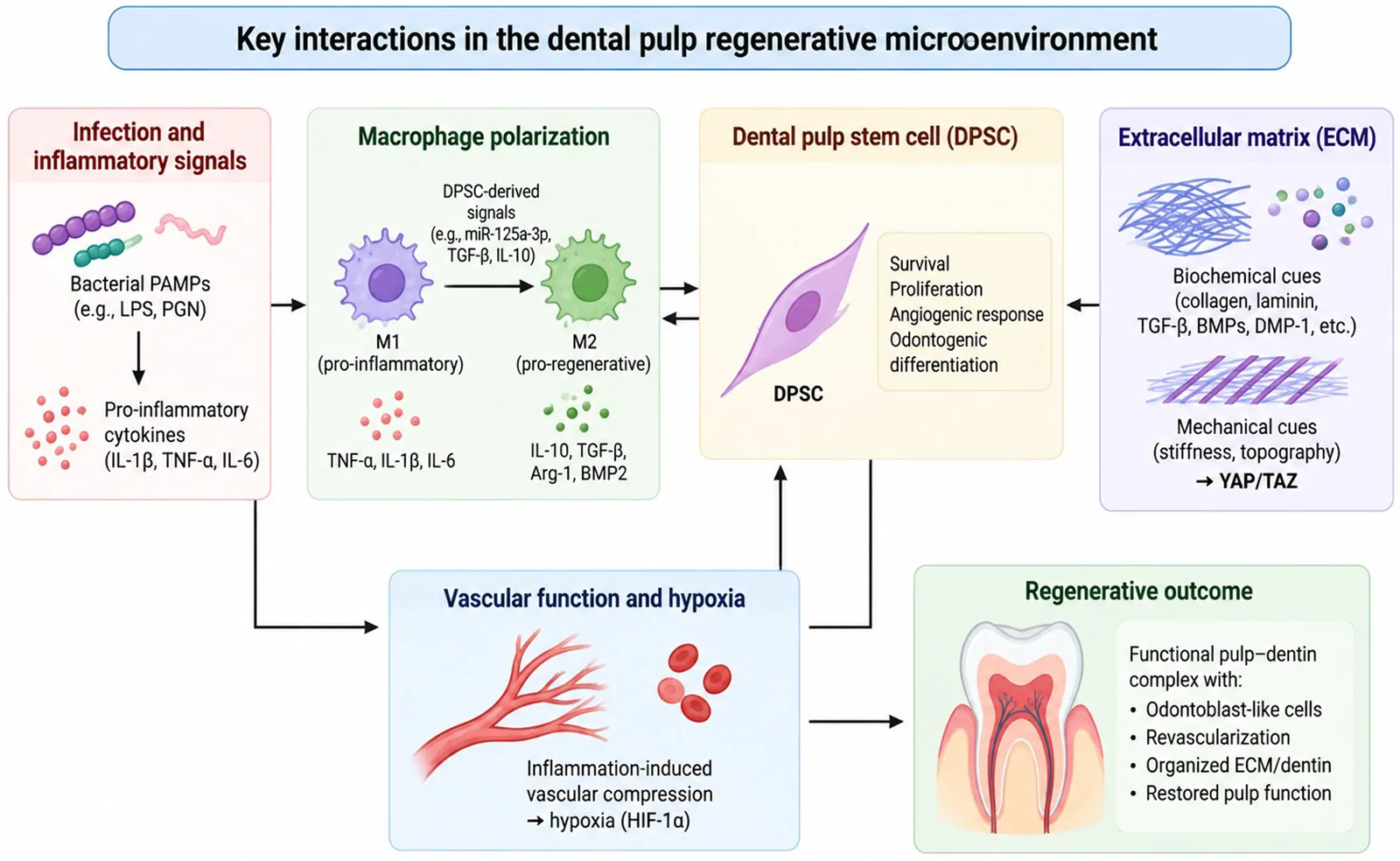
Anatomical confinement of the dental pulp conerts a defensive inflammatory response into a self-amplifying cascade. **(A)** Bacterial antigens and metabolic by-products from the overlying caries lesion and biofilm diffuse through dentinal tubules into the pulp. The boxed region in the tooth schematic is shown at higher magnification on the right to illustrate the dentin–pulp interface and the diffusion of microbial products through dentinal tubules. Because the pulp is enclosed within rigid, mineralized dentin walls, this inflammatory trigger arises within an anatomically confined space with minimal capacity for lymphatic drainage. **(B)** This confinement drives a self-amplifying vascular cascade: vascular leakage and immune-cell recruitment increase interstitial pressure within the confined pulp chamber; rising pressure compresses the surrounding vasculature; vascular compression produces blood stasis and hypoxia; and, if the stimulus is not resolved, sustained hypoxia may progress to pulpal necrosis, illustrating the transition from a defensive pathogen-clearing response to self-amplification and tissue damage. [Figure assembled using BioRender.].

The identity of the invading microorganism, not merely its presence, shapes the specific pattern of signaling activated in host cells. Relevant microorganisms include cariogenic and periodontal pathogens, such as *Streptococcus mutans* and *Porphyromonas gingivalis*, as well as opportunistic secondary invaders associated with persistent or post-treatment endodontic infections, including *Enterococcus faecalis* and *Candida albicans*. These microorganisms engage different combinations of MAPK, TGF-β/SMAD, and NF-κB signaling in dental pulp stem cells, producing microorganism-specific transcriptional and cytokine signatures rather than one stereotyped inflammatory program (Figure 2) (15). These pathogen-specific signaling networks are particularly evident in DPSCs, whose transcriptional responses vary according to the infecting microorganism, indicating that the regenerative potential of the pulp is influenced not only by the intensity of inflammation but also by microbial composition (16). At a broader level, Gram-positive organisms, largely via lipoteichoic acid, preferentially elicit IL-12 and TNF-α, whereas Gram-negative anaerobes and their lipopolysaccharide (LPS) preferentially induce IL-6, IL-1β, and IL-8 (15). This distinction parallels the clinical pattern of Gram-positive dominance in early lesions giving way to Gram-negative anaerobic dominance as caries deepens.

**Figure 2:**
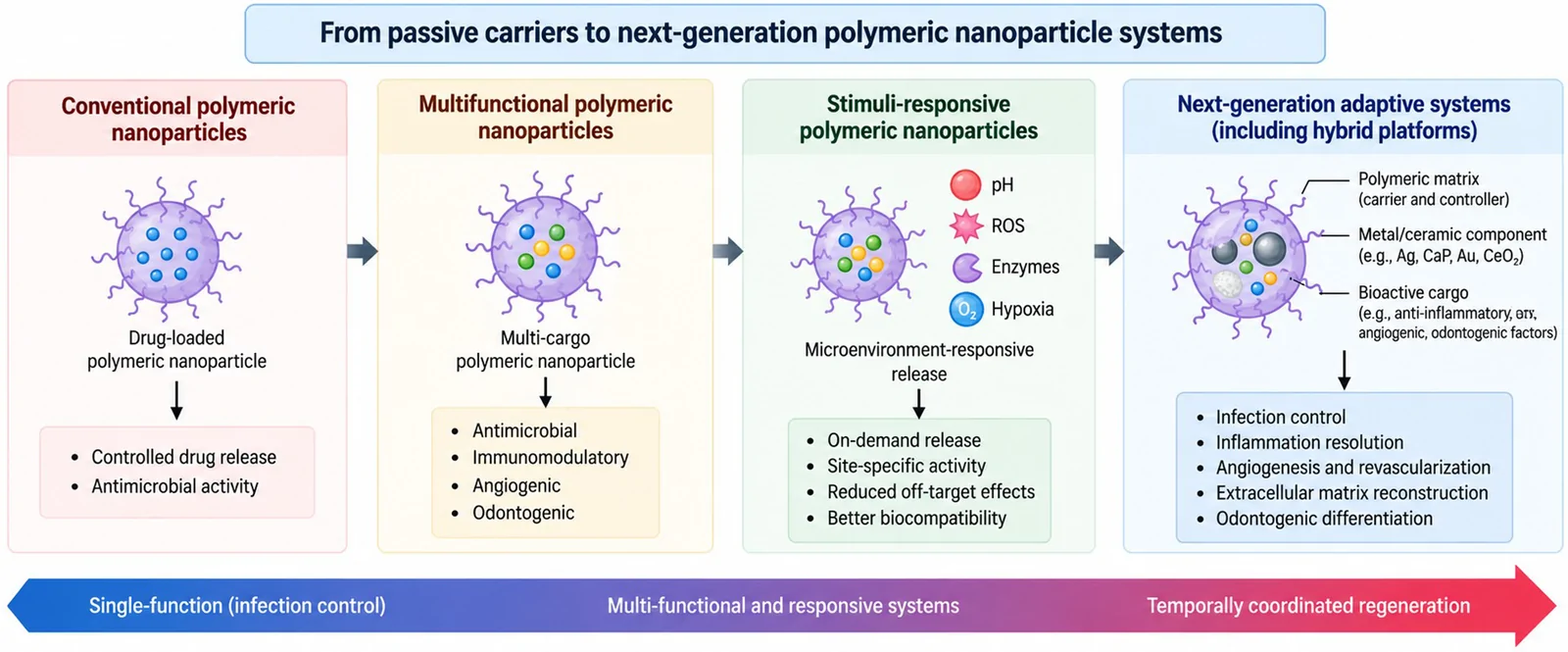
Pathogen-specific signaling networks in DPSCs regulate the pulp's inflammatory and regenerative gene program. Relevant microorganisms include cariogenic and periodontal pathogens, such as *Streptococcus mutans* and *Porphyromonas gingivalis*, as well as opportunistic secondary invaders associated with persistent or post-treatment endodontic infections, including *Enterococcus faecalis* and *Candida albicans*. These microorganisms engage DPSCs via pathogen-associated molecular patterns (PAMPs), activating an interconnected signaling network involving MAPK (p38/ERK/JNK), TGF-β/SMAD (SMAD2/3), and NF-κB (p65/p50), with extensive crosstalk between these pathways. The resulting pathogen-specific transcriptional program and cytokine signature regulate inflammation- and regeneration-associated genes, thereby influencing the pulp's overall regenerative potential. [Figure assembled using BioRender.].

Critically, recent evidence indicates that the odontogenic outcome of inflammatory stimulation depends not only on the induction of inflammatory signalling, but also on the ability of dental pulp cells to complete autophagic degradation. Autophagic flux, defined by autophagosome–lysosome fusion and subsequent lysosomal degradation, appears to function as a concentration-dependent regulatory mechanism. Low-concentration LPS promotes complete autophagic flux in human dental pulp cells and enhances odontogenic differentiation and mineralisation. In contrast, high-concentration LPS induces autophagosome formation but impairs autophagosome–lysosome fusion, as indicated by concurrent p62 accumulation, thereby suppressing the odontogenic programme. Importantly, restoration of autophagic flux reverses this inhibitory effect, suggesting that preservation of flux integrity is required to maintain the regenerative capacity of dental pulp cells under inflammatory stress (16). This concentration-dependent duality provides a mechanistic basis for why the intensity of inflammatory stimulation, rather than its mere presence, may influence whether the pulp retains its regenerative capacity or shifts toward a less reparative state: preservation of complete autophagic flux supports odontogenic differentiation, whereas disruption of autophagosome–lysosome fusion under stronger LPS stimulation suppresses the odontogenic programme (16).

### 3.2 Immune regulation and macrophage polarization

Recent work has begun to identify the specific molecular circuits that drive the M1-to-M2 shift, and DPSCs appear to be active players in this process rather than passive recipients of macrophage signals. One example is the transcription factor ATF3. Its expression decreases sharply in inflamed pulp tissue, and restoring it promotes macrophage polarization toward M2 by binding directly to the WNT4 promoter. WNT4 expression increases, CD86 and iNOS (M1 markers) decrease, and CD206 and Arg-1 (M2 markers) increase. This links a single transcription factor to both the macrophage phenotype and, through WNT4, the osteogenic behavior of nearby DPSCs (17). Importantly, this relationship does not conform to a simple M1-detrimental/M2-reparative dichotomy. M1 macrophages can act as pro-angiogenic partners to DPSCs: at low density, the IL-8 they secrete drives DPSCs to release VEGF via CXCR1/2-MAPK/ERK-HIF-1α signaling, producing vascular sprouting comparable to that observed with M2 co-culture. At higher M1 macrophage densities, however, this pro-angiogenic effect is reversed, with increased cytotoxicity and vascular regression (18). Thus, the effect of M1 macrophages is context-dependent, with low numbers supporting angiogenesis and higher numbers producing the opposite effect (18).

The relationship also runs in both directions. DPSCs release small extracellular vesicles carrying miR-125a-3p, which polarizes macrophages toward M2 by directly targeting IKBKB and suppressing TLR4/NF-κB signaling. These newly polarized M2 macrophages then secrete BMP2, which activates BMPR1–SMAD1/5/9 signaling in DPSCs and promotes their odontogenic differentiation (19). This reciprocal signaling allows DPSCs to reshape the local immune environment, which in turn feeds back on their odontogenic differentiation (19).

### 3.3 Hypoxia and vascularization

The inflammatory response that initially protects the pulp also creates one of the greatest barriers to regeneration: hypoxia. Because the pulp is enclosed within rigid dentin, inflammatory edema rapidly increases intrapulpal pressure and compresses the microvasculature, restricting blood flow and reducing oxygen delivery (20). Rather than being a passive consequence of inflammation, this hypoxic environment actively reshapes cellular behavior through oxygen-sensitive signaling pathways. Among these, hypoxia-inducible factor-1α (HIF-1α) is a key regulator linking oxygen availability to inflammatory, angiogenic, and reparative responses in the pulp (15).

Recent evidence shows that hypoxia alone is sufficient to amplify pulpal inflammation. In human dental pulp fibroblasts, reduced oxygen stabilizes HIF-1α, which activates NF-κB signaling and promotes ASC oligomerization, leading to assembly of the NLRP3 inflammasome. This results in caspase-1 activation and increased secretion of the pro-inflammatory cytokines IL-1β and IL-18, indicating that hypoxia does not simply accompany inflammation but actively sustains it. When bacterial LPS is present, HIF-1α expression and inflammasome activation become even more pronounced, demonstrating that microbial infection and hypoxia reinforce one another through a shared HIF-1α–NF-κB–NLRP3 signaling axis (15, 21). Conversely, silencing HIF-1α suppresses NF-κB activation, reduces inflammasome activity, and limits cytokine production, identifying HIF-1α as an upstream regulator of inflammatory amplification rather than merely a marker of tissue hypoxia (15, 21).

Hypoxia, however, is not exclusively detrimental. HIF-1α also regulates the angiogenic response required for tissue regeneration by stimulating vascular endothelial growth factor (VEGF) expression and promoting endothelial cell recruitment (22). The regenerative outcome therefore depends on the duration and severity of oxygen deprivation. Transient or moderate hypoxia can stimulate vascular remodeling and restore oxygen delivery, creating conditions that support DPSC survival and odontogenic differentiation (23). In contrast, prolonged hypoxia maintains inflammasome activation, perpetuates cytokine release, and further compromises microcirculation, establishing a self-reinforcing cycle of inflammation, vascular dysfunction, and tissue damage (24). Thus, successful regeneration requires not the complete elimination of hypoxic signaling, but restoration of oxygen homeostasis before hypoxia shifts from an adaptive response to a pathological driver of chronic inflammation (25).

### 3.4 Extracellular matrix as a biological niche

As inflammatory and hypoxic conditions evolve, the integrity and composition of the pulp ECM become additional determinants of regenerative outcome. The ECM of the dentin–pulp complex is not an inert filler occupying the space between cells; it behaves as a physiological depot that sequesters growth factors and matrix molecules such as TGF-β, BMP, and a family of non-collagenous proteins during development. These molecules are released gradually during matrix degradation or remodeling, thereby altering the local availability of signals involved in pulp repair (26). This dual identity, as a structural scaffold and biochemical reservoir, allows the postnatal pulp to maintain a population of quiescent progenitor/stem cells within discrete niches. Each niche is composed of heterologous cell types, ECM, and soluble factors that hold resident stem cells at rest until injury releases the cues needed for their proliferation and differentiation into odontoblast-like cells (26).

The instructive capacity of this niche is carried by specific molecules rather than by matrix bulk alone. Dentin matrix protein-1 (DMP-1) acts as a morphogen on undifferentiated mesenchymal cells at the dentin–pulp interface, inducing cytodifferentiation of DPSCs into odontoblasts and driving deposition of dentin-like tissue when presented on an insoluble collagenous substrate. However, the same molecule loses this activity when immobilized on an inert membrane rather than on native dentin, indicating that its bioactivity depends on the surrounding insoluble matrix and not on the peptide in isolation (26). Related non-collagenous proteins, including dentin sialoprotein/phosphoprotein, bone sialoprotein, and MEPE, work on a comparable principle. Several of them can independently trigger a reparative dentinal bridge when implanted into exposed pulp, recruiting local cells that proliferate and differentiate into osteoblast- and odontoblast-like phenotypes (26). Taken together, these findings indicate that the ECM does not merely support odontogenic differentiation; its molecular composition actively influences odontogenic cell fate.

Recognizing this, a growing body of tissue-engineering work has tried to recreate, rather than merely support, the native niche, using decellularized extracellular matrix (dECM) and treated dentin matrix (TDM) as scaffolds. These scaffolds retain much of the original tissue's structural proteins (type I/III collagen, laminin, fibronectin), together with residual bioactive cargo such as TGF-β, DMP-1, and dentin sialoprotein, once the immunogenic cellular components have been removed (27, 28). Because these matrices preserve the vascular architecture and growth-factor content of the original pulp, conditioned media and hydrogels derived from them enhance dental pulp stem cell proliferation, migration, and odontogenic differentiation. They also support pulp-like tissue formation *in vivo*, effectively reconstituting, at least in part, the niche that inflammation and hypoxia had disrupted (27, 28). TDM in particular behaves as a slow-release reservoir for dentinogenic proteins and has already been used as a pulp-capping and direct-capping material. This underscores that even a devitalized, chemically treated matrix retains enough of its native chemistry to instruct regeneration (27, 28).

The ECM's role as a reservoir extends naturally to the retention of therapeutic cargo introduced from outside the tissue. Extracellular vesicles loaded into ECM-based carriers (dECM, TDM, collagen, fibrin) or ECM-bioinspired hydrogels (GelMA, alginate, chitosan, self-assembling peptides) are protected from rapid clearance and degrade in step with the surrounding matrix. This sustains local delivery of pro-angiogenic and odontogenic signals for long enough to support angiogenesis, immunomodulation, and dentin-bridge formation (28). This principle, that the matrix determines not only which signals are present but also how long they persist and where they act, is directly relevant to the premise of this review. A polymeric nanoparticle engineered to integrate with, rather than simply diffuse through, the ECM niche may be better positioned to reproduce the coordinated, multi-signal instruction that natural regeneration requires.

Beyond its molecular cargo, the physical state of the ECM, intact or degraded, soft or stiff, hydrated or desiccated, carries its own instructive weight, since resident and recruited stem cells read matrix mechanics almost as readily as they read cytokine gradients. It is this convergence of biochemical and biomechanical cues, layered onto the inflammatory and hypoxic history of the tissue, that ultimately steers stem-cell fate toward an odontoblast-like, osteogenic, or fibrous outcome, as discussed next.

### 3.5 Stem-cell fate is shaped by the microenvironment

The inflammatory, hypoxic, immune, and extracellular-matrix changes described above ultimately converge on the behavior and fate of resident and recruited stem-cell populations. DPSCs are not a homogeneous reservoir waiting for one instructive cue; their fate reflects the combined, and often conflicting, signals present in the microenvironment at the moment of injury (29).

Four signaling systems sit at the center of this decision: TGF-β, Wnt, Notch, and FGF. They act individually and, critically, through crosstalk to specify whether a DPSC commits to an odontoblast/osteoblast-like lineage or remains undifferentiated (29). None behaves as a simple binary switch. TGF-β1 illustrates this well: short-term priming with TGF-β before osteogenic induction upregulates RUNX2, osterix, DSPP, and DMP-1 and accelerates mineralization. Yet continuous or late-stage TGF-β exposure suppresses terminal mineralization by promoting Runx2 degradation, so the same ligand can prime or block the same lineage depending purely on the timing of exposure (29).

Wnt/β-catenin signaling shows an equivalent duality, but at the level of magnitude rather than time. Low-to-moderate activation via Wnt3a, Wnt10a, or small-molecule GSK-3β antagonists such as tideglusib and CHIR99021 drives reparative dentinogenesis and mineralized-nodule formation, whereas excessive or sustained pathway activation produces pathological, hypomineralized dentin and risks pulp obliteration rather than physiological repair (29, 30). Notch and FGF add further context dependence. Notch can either promote or restrain osteogenic commitment depending on ligand identity and on whether the ligand is presented in soluble or immobilized form, while bFGF expands the progenitor pool during an early window but actively restrains terminal odontoblastic differentiation once exposure is prolonged (29). The microenvironment, in effect, does not simply switch signaling on; it sets the dose, duration, and presentation of the signal, and DPSCs read all three.

The ECM discussed above is not a passive stage for this signaling but a co-determinant of it. DPSCs are markedly sensitive to substrate stiffness: culturing DPSCs on progressively stiffer matrices approximating mineralized dentin upregulates osteo/odontogenic markers through YAP/TAZ-dependent mechanotransduction, whereas very low stiffness favors a more quiescent or fibroblastic phenotype (29). Yet this mechanosensing is not absolute. In the absence of a directional stiffness gradient, DPSCs migrate unpredictably between soft and stiff substrates, independent of myosin-II activity or YAP nuclear translocation, using lamellipodia-driven mesenchymal migration. They still retain the capacity to differentiate into odontoblasts once the appropriate biochemical cues arrive (31). This indicates that mechanical cues bias, rather than dictate outright, the differentiation decision and that biophysical and biochemical signals must converge before a stable fate commitment occurs.

This convergence is complicated further because the pulp does not contain one uniform DPSC population but several, each occupying a distinct niche with a distinct default behavior. Subpopulations co-expressing STRO-1 and CD146 and negative for CD271 are highly clonogenic and multipotent, whereas CD271-positive, short-telomere subpopulations are largely restricted to adipogenic or neurogenic fates. Cells residing in the perivascular niche likewise behave differently from those in the sub-odontoblastic layer or apical papilla, reflecting distinct developmental origins within the neural crest lineage (32). Because only a minority of clonogenic DPSCs are capable of extensive proliferation and multilineage differentiation, the apparent “regenerative response” of the pulp to any given microenvironmental cue is really the aggregate output of several coexisting subpopulations responding differently to the same signal, rather than a uniform response of one cell type (32).

Migration behavior tracks this heterogeneity closely. Cells that emerge first from injured or explanted pulp tissue (“leader” cells) migrate as single cells under combined biochemical and mechanical stimulation and differentiate rapidly. In contrast, cells that emerge later (“follower” cells) remain closer to the native tissue, proliferate preferentially, and preserve a self-renewing, less-differentiated state. This implies that the timing and mode of a cell's migration out of its niche is itself part of the fate decision, not merely a downstream consequence of it (31).

Finally, the inflammatory intensity described in Section 3.1 feeds directly back into this same decision node through Wnt/NF-κB crosstalk. Low-dose, transient inflammatory stimulation activates NF-κB in a manner that synergizes with Wnt signaling to promote odontogenic gene expression (RUNX2, DSPP) and self-renewal, effectively repurposing an early defensive signal into a reparative one. Sustained, high-dose inflammatory exposure instead drives NF-κB to upregulate the Wnt antagonist DKK-1, suppressing β-catenin nuclear translocation, blocking DSPP/DMP-1 expression, and shifting the same cell toward pyroptosis and stemness exhaustion rather than repair (30).

Taken together, inflammation, hypoxia, matrix state, and stem-cell fate are not sequential, isolated events but a single interlocking circuit. The same microenvironmental variables that determine whether a macrophage adopts an M1 or M2 phenotype and whether the matrix is degraded or retained as a signaling reservoir ultimately influence whether the DPSC exposed to those conditions becomes an odontoblast, an osteoblast, a quiescent progenitor, or a cell that fails to differentiate at all. These interconnected relationships are summarized schematically in Figure 3, highlighting how inflammatory, immune, vascular, and extracellular-matrix cues converge to regulate DPSC behavior and regenerative outcome.

**Figure 3:**
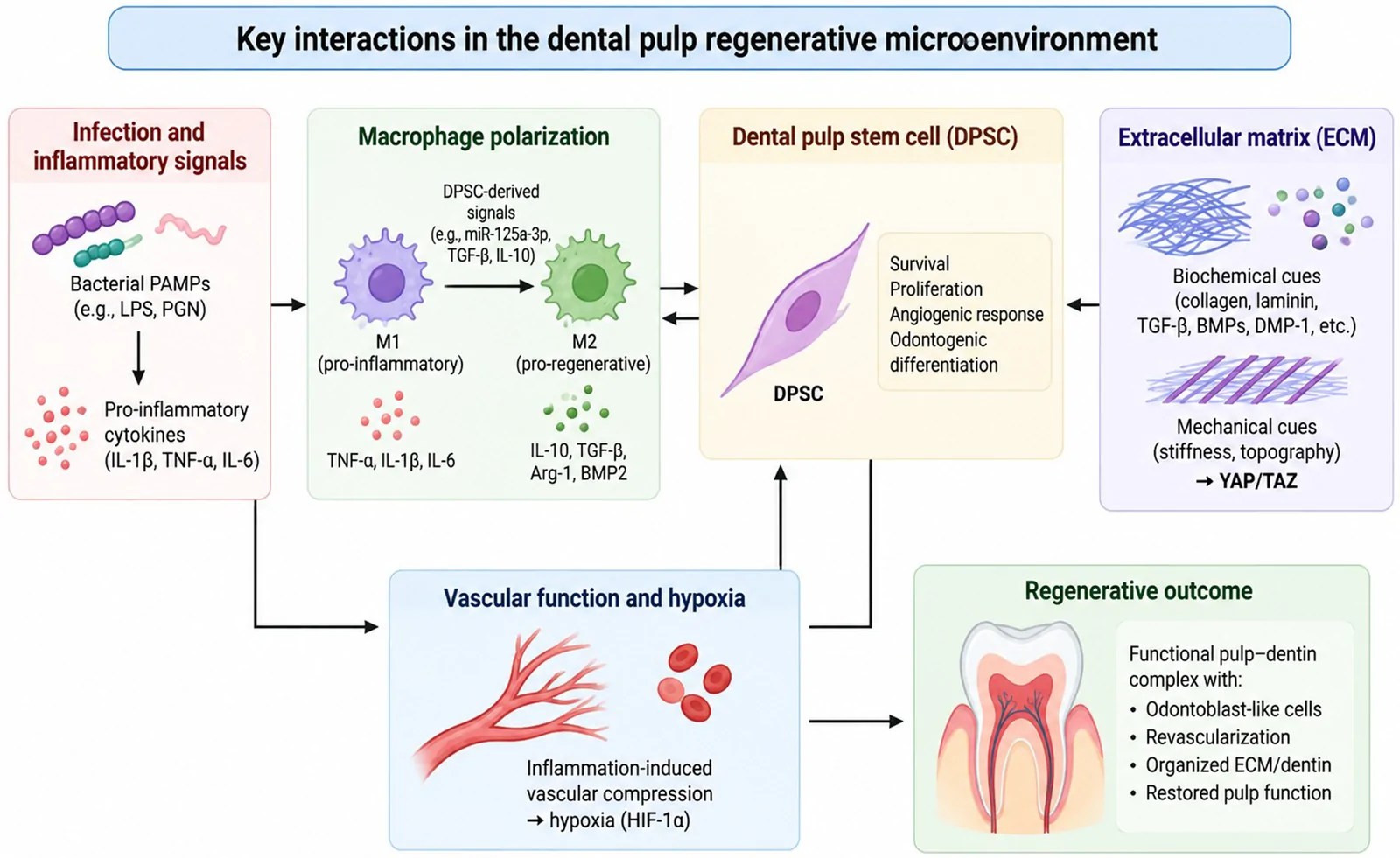
Key interactions in the dental pulp regenerative microenvironment. Infection-associated inflammatory signals influence macrophage phenotype and vascular function, while hypoxia, extracellular-matrix biochemical and mechanical cues, and reciprocal macrophage–DPSC signaling collectively regulate DPSC survival, proliferation, angiogenic activity, and odontogenic differentiation. The balance among these interconnected signals ultimately shapes whether a functional pulp–dentin complex is regenerated. [Figure assembled using BioRender.].

## 4 From passive drug carriers to smart polymeric nanoparticles

To maintain continuity between the biological challenges described in Section 3 and the corresponding material strategies, the following subsections are organized around the same major therapeutic targets: infection and inflammation, macrophage regulation, vascular dysfunction and hypoxia, extracellular-matrix reconstruction, and stem-cell fate. Direct evidence for polymeric nanoparticles capable of regulating the pulp microenvironment remains limited, while related nanoscale and biomaterial systems provide complementary mechanistic and engineering principles that can inform future polymeric nanoparticle design.

If stem-cell fate is shaped by the interlocking state of inflammation, hypoxia, immune polarization, and extracellular-matrix architecture, then the therapeutic objective must extend beyond antimicrobial drug delivery. Material architecture alone can influence regenerative behavior: bilayered PLGA scaffolds with different pore sizes produced distinct odontoblastic responses from DPSCs on opposing surfaces of the same construct (33). Likewise, the release kinetics of nanoparticle-associated signals, including dexamethasone and TGF-β1 delivered through chitosan-based systems, can determine whether SCAP migrate, differentiate toward an odontoblast-like phenotype, or remain relatively undifferentiated (34, 35).

A material introduced into the root canal is therefore not merely required to eliminate microorganisms or release a therapeutic molecule. Ideally, it should intervene in the biological circuit that determines whether inflammation resolves, vascularization is restored, the ECM is reconstructed, and resident or recruited stem cells undergo odontogenic rather than fibrous differentiation. Evidence from bone-regenerative systems further indicates that nanoparticle surface chemistry, degradation products, and ion release can alter macrophage activation and thereby influence osteogenesis and angiogenesis. However, the direct translation of these findings to the confined and infection-prone pulp environment remains incompletely established.

Viewed against these requirements, first-generation antibiotic-loaded carriers addressed only part of the regenerative challenge. Although high local antibiotic concentrations can suppress intracanal infection, they can also compromise SCAP survival (36). Subsequent systems have attempted to narrow this disinfection–biocompatibility gap through concentration-sparing antibiotic-eluting nanofibers (37), sustained-release carriers with controlled burst and plateau phases (38), and stimuli-responsive platforms that release cargo in response to pathological cues such as acidic pH, proteolytic enzymes, reactive oxygen species, or temperature changes (39). The evolution of polymeric nanotechnology in regenerative endodontics can therefore be understood as a transition from passive drug carriage toward active regulation of the pulp microenvironment.

### 4.1 Nanoparticles that interrupt the infection–inflammation cascade

Section 3.1 established that persistent microbial challenge sustains inflammatory signaling, whereas prolonged exposure to high concentrations of intracanal antibiotics can damage the stem-cell populations required for regeneration. The therapeutic challenge is therefore not simply to maximize antimicrobial exposure, but to achieve sufficient biofilm control while minimizing unnecessary contact between cytotoxic agents and DPSCs or SCAP. Stimuli-responsive delivery systems offer a potential solution by retaining their cargo under relatively quiescent conditions and increasing release in response to infection-associated cues such as acidic pH, bacterial enzymes, inflammatory proteases, or elevated reactive oxygen species.

Proof of this principle has been demonstrated most clearly outside endodontics. Ding et al. coated silver-loaded mesoporous silica nanoparticles with poly-L-glutamic acid, producing increased silver release in the presence of the bacterially secreted V8 protease and lower release in its absence (40, 41). Although this system was not developed for the root canal and was not a purely polymeric nanoparticle, it illustrates how an infection-associated enzyme can be converted into a local release trigger.

More direct evidence has emerged in regenerative endodontics. Xie et al. developed an inflammation-responsive gold-nanocluster hydrogel containing ciprofloxacin and metronidazole. Exposure to infected root-canal interstitial fluid promoted hydrogel swelling and protease-associated degradation, resulting in prolonged antimicrobial release while supporting stem-cell proliferation, migration, adhesion, and mineralization (42). This composite system is particularly relevant because it links antimicrobial responsiveness with regenerative-cell compatibility. However, its gold-nanocluster and hydrogel composition places it within a broader class of responsive nanocomposites rather than conventional polymeric nanoparticles alone.

### 4.2 Nanoparticles that regulate macrophage polarization

Macrophage regulation in regenerative endodontics should not be reduced to simply eliminating M1-like macrophages or maintaining a permanent M2 phenotype. As discussed in Section 3.2, an early pro-inflammatory response can still support microbial defense, debris clearance, and angiogenic signaling. When this response becomes excessive or persistent, however, sustained macrophage activation can amplify inflammatory signaling, oxidative stress, vascular dysfunction, and stem-cell injury. The therapeutic aim is therefore to retain sufficient early innate immune activity while encouraging a timely shift toward macrophage states that support inflammation resolution, extracellular-matrix remodeling, angiogenesis, and odontogenic differentiation (43–45).

Direct evidence that a nanoscale system can influence this transition in inflamed pulp tissue comes from an antioxidase-mimicking carbon-dot nanozyme incorporated into injectable GelMA (46). The C-NZ/GelMA nanocomposite scavenged excessive reactive oxygen and nitrogen species in LPS-induced pulpitis, with fewer CD86-positive macrophages, an early increase in CD206-positive macrophages, and reduced TNF-α and IL-6. Notably, the CD206-positive response declined as inflammation resolved rather than remaining persistently elevated. In rat pulpitis models, these changes were accompanied by vascularized pulp-like tissue and reparative dentin with organized tubular features (46). This temporal pattern argues against permanent M2 dominance and instead supports controlled progression through inflammatory and reparative phases.

The surrounding matrix can also shape macrophage behavior. An injectable decellularized dental-pulp ECM hydrogel increased Arg-1, CD206, IL-10, TGF-β1, TGF-β3, and BMP2 while reducing iNOS. Conditioned medium from these macrophages subsequently enhanced SHED migration, proliferation, alkaline phosphatase (ALP) activity, and odontogenic differentiation (47). *In vivo*, the hydrogel increased the early M2/M1 balance and supported reparative dentin formation after direct pulp capping. However, the phenotype did not fully reproduce classical IL-4-induced M2 polarization, with both CD80 and CD206 increasing in some analyses, and the responsible matrix-derived signals remain unclear (47). The response is therefore better interpreted as context-dependent macrophage programming than as a binary M1-to-M2 switch.

Immunoregulatory cargo provides another route. DPSC-derived exosomes retained within a chitosan hydrogel increased CD206, reduced CD86, and suppressed inflammatory signaling in a mouse periodontitis model (48). miR-1246 contributed to this response, as its inhibition weakened macrophage transition, reactivated NF-κB p65 and p38 MAPK signaling, and reduced protection against epithelial injury and alveolar-bone loss (48). Although this was a periodontal rather than pulp model, and the active vesicles were exosomes rather than polymeric nanoparticles, it provides a useful precedent for polymeric carriers designed to protect and release regulatory RNA with controlled loading and release.

Polymeric nanoparticles may also be designed to accumulate preferentially in inflamed tissue. Tasquinimod-loaded PLGA nanoparticles coated with TLR4-rich macrophage membranes accumulated in diseased colonic tissue and reduced S100A9, NF-κB p65, STAT3, and pro-inflammatory cytokines (49). The gastrointestinal model neither demonstrated pulp regeneration nor established an M1-to-M2 transition, but it shows that macrophage-membrane functionalization can combine inflammatory targeting, sequestration of inflammatory mediators, and local delivery of an NF-κB-dampening cargo. Translation to the root canal would require validation under its restricted diffusion, bacterial biofilm, and limited vascular access (49).

Macrophage regulation may also influence regeneration beyond inflammatory suppression. M2 macrophage-derived exosomes were taken up by DPSCs and endothelial cells and enhanced DPSC migration, proliferation, odontogenic and neurogenic differentiation. They also enhanced endothelial migration, tube formation, VEGF expression, and CD31-positive vascular-network formation (50). However, their active proteins and RNAs were not identified, and the *in vivo* experiments used a subcutaneous rather than an orthotopic pulp model (50). These vesicles therefore provide a biological guide for identifying future nanoparticle cargo rather than an established regenerative nanotherapy.

Together, these studies point to three complementary routes for macrophage-directed nanotherapy: redox correction, delivery of immunoregulatory cargo, and preferential targeting of inflamed tissue (46, 48, 49). Direct evidence for conventional polymeric nanoparticles in regenerative endodontics nevertheless remains limited; the current evidence spans nanozyme–hydrogel composites, ECM-derived hydrogels, extracellular-vesicle systems, and a polymeric platform tested outside the pulp (46–50). Future studies should therefore move beyond CD86, iNOS, CD206, and Arg-1 and connect time-dependent immune changes with bacterial clearance, cytokine profiles, DPSC/SCAP survival and differentiation, endothelial recruitment, organized dentin formation, and restoration of functional pulp-like tissue (46, 47, 50). The broader design objective is not permanent macrophage polarization, but a self-limiting immune response in which early defense gives way to a regenerative phase as the local pathological burden resolves. This remains a design principle derived from the available evidence rather than a capability already demonstrated by a single polymeric nanoparticle system.

### 4.3 Nanoparticles that correct hypoxia and vascular dysfunction

The vascular problem in regenerative endodontics is not simply a shortage of VEGF. As discussed in Section 3.3, the regenerating pulp must establish a functional microcirculation through a narrow apical entry while cells within the canal face limited oxygen and nutrient delivery (51, 52). Hypoxia can initially stimulate angiogenesis through HIF-1α-dependent VEGF expression (51), but persistent oxygen deprivation may sustain the pathological microenvironment when vascular recovery is delayed. The therapeutic objective is therefore to accelerate formation of a functional vascular network and restore oxygen homeostasis rather than maintain hypoxic signaling indefinitely.

One approach used injectable nanofibrous spongy microspheres (NF-SMS) assembled from a star-shaped polymer to deliver hypoxia-primed hDPSCs (51). The nanofibrous architecture supported cell attachment and growth, while hypoxic preconditioning activated HIF-1α and increased endogenous VEGF expression. Hypoxia-primed hDPSC/NF-SMS constructs subsequently generated pulp-like tissue with greater vascularity than normoxia-preconditioned constructs, including in an *in situ* pulp-regeneration model (51). Rather than delivering VEGF directly, this strategy used transplanted cells as local sources of angiogenic signaling.

A more direct nanocarrier strategy used a multifunctional pH-sensitive polymeric non-viral vector to deliver plasmid VEGF (pVEGF) to DPSCs (52). The carrier incorporated functions for cellular uptake, endosomal escape, and nuclear localization, addressing sequential intracellular barriers to gene delivery. VEGF expression increased more than eightfold after transfection, with enhanced endothelial migration and vascular-like tube formation (52). In a full-length human tooth-root model, pVEGF-transfected DPSCs generated abundant blood vessels throughout the canal and increased regenerated tissue from 32.5% with unmodified DPSCs to 68.7% (52). Importantly, pH responsiveness was used primarily to facilitate intracellular trafficking and endosomal/lysosomal escape. This extends the concept of responsive delivery beyond controlling extracellular release: nanoparticle responsiveness can also determine where cargo becomes available after cellular uptake.

Vascular support can also be incorporated into multifunctional antimicrobial materials. AgNPs combined with DPSCs in a photosensitive GelMA hydrogel provided antibacterial activity while maintaining >90% cell viability at the selected AgNP concentration. DPSC-containing constructs also promoted capillary-like structure formation and expression of CD31, VEGF, and vWF (53). Although these angiogenic findings remain *in vitro* and cannot establish restoration of pulpal perfusion, they show that infection control can be designed alongside preservation of vascular cell behavior rather than treated as an independent objective.

Together, these approaches act at different levels of the vascular problem: hypoxic preconditioning can recruit endogenous HIF-1α–VEGF signaling (51), polymeric nanocarriers can deliver genetic instructions for local VEGF production (52), and multifunctional nanocomposites can combine infection control with preservation of angiogenic behavior (53). Their purpose, however, should not be sustained activation of hypoxia-responsive or pro-angiogenic pathways.

A more advanced strategy would couple angiogenic activity to the changing pulp environment. For example, hypoxia- or ROS-responsive polymeric carriers could adjust pro-angiogenic cargo delivery as vascular conditions change, potentially alongside pH- or enzyme-responsive mechanisms linked to infection and inflammation. Such adaptive vascular control has not yet been demonstrated by the endodontic systems discussed here, but represents a logical progression from sustained angiogenic delivery toward microenvironment-responsive regulation of pulp revascularization.

### 4.4 Nanoparticles that reconstruct or mimic the extracellular-matrix niche

Nanomaterial-mediated reconstruction of the pulp niche can combine nanoscale structural mimicry with restoration of matrix-associated biochemical signaling. Self-assembling RAD/Dentonin hydrogels form a β-sheet nanofibrous network containing the ECM-derived RGD and SGDG motifs of Dentonin, supporting DPSC adhesion, migration, and proliferation while enhancing odontogenic differentiation and mineral deposition (54). A complementary strategy integrates calcium-phosphate nanoparticles carrying DMP1- or BMP2-encoding plasmids with treated human dentin scaffolds. Cellular uptake of these nanoparticles provides additional dentinogenic signaling after exposure to factors retained within the dentin matrix, promoting odontogenic differentiation and polarization of DPSCs toward an odontoblast-like phenotype at the dentin–pulp interface (55).

Natural nanoscale vesicles provide another instructive model: odontogenically conditioned DPSC-derived exosomes transfer regulatory miRNAs that enhance odontogenic differentiation through TGF-β1/Smad signaling, including downregulation of LTBP1 (56). Together, these approaches move beyond passive scaffold filling toward nanomaterials that reproduce structural, matrix-associated, and intercellular signals of the dentin–pulp niche. For polymeric nanoparticle design, the important next step is to integrate these functions within a single controllable platform rather than supplying individual ECM signals in isolation.

### 4.5 Nanoparticles that direct stem-cell fate

As summarized in Table 1, polymeric nanosystems can influence dental stem-cell fate by controlling the presentation of differentiation-regulating signals rather than simply serving as passive drug carriers. Temporally controlled dexamethasone release from chitosan nanoparticles enhanced SCAP odontogenic differentiation (34), while sustained delivery of bioactive TGF-β1 from a chitosan nanoparticle–scaffold system promoted SCAP migration, viability, biomineralization, and expression of odontogenic markers (35). Chitosan nanogels carrying GM-CSF provide a further route for fate regulation, increasing DSPP, DMP1, and OCN expression in hDPSCs, although this evidence remains limited to an *in vitro* model (60). Together, these systems show that nanoparticle-mediated fate control can be achieved through temporal, sustained, or cytokine-based presentation of instructive biochemical cues.

**Table 1:** Representative polymeric nanoparticle systems and complementary nanoscale or biomaterial strategies relevant to regulation of the pulp microenvironment.

Target	Platform	Cargo/active component	Mechanism	Key regenerative finding	Evidence category	Ref.
Infection control	Inflammation-responsive gold nanocluster hydrogel (DA@Au NCs)	Ciprofloxacin + metronidazole	Root-canal infection interstitial fluid induces hydrogel swelling; increased fluid and internal proteases trigger degradation and DA release; cross-linking time regulates degradation duration	Antimicrobial activity while supporting stem-cell proliferation, migration, adhesion and mineralization	Direct endodontic nanocomposite (non-polymeric NP)	(42)
Infection control with reduced antibiotic exposure	Polydioxanone (PDS) nanofibers	Metronidazole + ciprofloxacin + minocycline	Localized, low-dose antibiotic delivery	Near-complete elimination of multispecies dentin biofilm, comparable to TAP	Direct *in-vitro* endodontic polymer-nanofiber evidence	(37)
Pulpitis/oxidative stress	Injectable GelMA hydrogel	Carbon dot nanozyme (C-NZ)	RONS scavenging + M2 macrophage polarization	Reduced oxidative stress and inflammation; promoted pulp regeneration and dentin repair	*In vitro* + *in vivo* rat pulpitis nanocomposite (non-polymeric nanozyme)	(46)
Dental pulp regeneration/odontogenesis	DPSC-derived small extracellular vesicles (DPSCs-sEV)	miR-125a-3p-enriched sEV	miR-125a-3p → IKBKB targeting → TLR/NF-κB suppression → M2 polarization → ↑ BMP2/BMP signaling	Reduced pro-inflammatory signaling; promoted M2 macrophage phenotype and odontogenic differentiation of DPSCs	*In vitro* + *in vivo* rat pulp-capping model; extracellular-vesicle precedent (non-polymeric)	(19)
Angiogenesis	Cell membrane-coated nano microspheres	Hypoxia-induced SHED pro-angiogenic factors	Sustained cytokine delivery	Enhanced vascularization and pulp regeneration	Direct *in-vivo* pulp-regeneration nano system (non-polymeric NP)	(58)
ECM/inflammatory niche	Self-assembling Fmoc-Phe3 hydrogel	Cerium-oxide nanoparticles + DMP-1	Antioxidant/anti-inflammatory regulation + dentinogenic signaling	Promoted pulp repair and reparative dentin	*In vitro* + *in vivo* pulp-capping nanocomposite (non-polymeric NP)	(59)
Odontogenic fate	Chitosan nanoparticles	Dexamethasone	Sustained controlled release	Enhanced SCAP odontogenic differentiation and biomineralization	Direct *in vitro* polymeric-NP evidence	(34)
Odontogenic fate	Chitosan nanoparticle + scaffold	TGF-β1	Sustained bioactive release	Enhanced SCAP migration and odontogenic differentiation	Direct polymeric-NP/scaffold evidence	(35)
Odontogenic fate	Chitosan nanogel	GM-CSF	Controlled cytokine delivery	Increased odontogenic differentiation markers in hDPSCs	*In vitro* polymeric nanogel	(60)
Stem-cell fate	Microporous GelMA–dextran construct	None; instructive architecture	YAP-associated mechanotransduction	Enhanced DPSC stemness and odontoblast-like differentiation	Biomaterial/mechanistic precedent (non-NP)	(57)

The evidence type distinguishes direct polymeric nanoparticle studies from polymer-containing nanocomposites and supporting mechanistic or design precedents.

Evidence from non-nanoparticle biomaterials provides a complementary mechanistic perspective on how material architecture can regulate stem-cell fate. An interconnected microporous GelMA–dextran construct increased nuclear YAP localization together with OCT4, NANOG, and SOX2 expression in hDPSCs (57). Under odontogenic conditions, the same architecture increased ALP activity and expression of RUNX2, OCN, OPN, and DSPP, supporting odontoblast-like differentiation (57). Although this construct is not itself a nanoparticle system, it identifies mechanotransduction as an additional design variable that could be integrated with polymeric nanocarriers. The next generation of fate-directed platforms should therefore move beyond maximizing differentiation markers toward coordinating the identity, concentration, duration, and physical context of instructive signals, so that odontoblast-lineage commitment is promoted without driving indiscriminate mineralization or non-pulpal tissue formation.

### 4.6 Role of polymeric components in hybrid organic–inorganic nanocarriers

Although this review focuses on polymeric nanoparticles, many emerging strategies in regenerative endodontics do not rely on polymers alone. Instead, polymer-containing matrices and carriers are increasingly combined with metallic or other inorganic nanomaterials to bring different functions into the same system (42, 53, 59). In these hybrid platforms, the polymer is more than a passive carrier. The inorganic component may provide antimicrobial, redox-modulating, mineralizing, or dentinogenic activity, while the polymeric component can help control cargo retention, degradation, and therapeutic release (41, 42, 53). This division of roles is particularly relevant in the pulp, where strong antimicrobial or mineralizing effects need to be achieved without compromising the cells required for regeneration.

Silver-containing systems provide a useful example. Although silver-based nanomaterials offer potent antimicrobial activity, their exposure must be balanced against the need to preserve DPSCs and other regenerative cell populations. Combining silver with a polymeric component provides one way of gaining greater control over when and how much of the antimicrobial agent is released. In the system reported by Ding et al., silver-loaded mesoporous silica nanoparticles were coated with poly-L-glutamic acid, and silver release increased in the presence of bacterially secreted V8 protease (41). Here, the silver provides antimicrobial activity, whereas the polymeric coating adds responsiveness to an infection-associated signal. A similar balance between antimicrobial activity and cell compatibility was demonstrated when AgNPs were incorporated into a photosensitive GelMA system containing DPSCs. Antibacterial activity was retained while DPSC viability remained high at the selected AgNP concentration (53).

Hybrid or composite systems containing ceramic components may serve a different purpose. Calcium-phosphate nanoparticles carrying DMP1- or BMP2-encoding plasmids, for example, have been combined with treated dentin scaffolds to enhance dentinogenic signaling and promote odontoblast-like differentiation at the dentin–pulp interface (55). Such systems illustrate how an inorganic component can contribute mineral-associated or dentinogenic properties while the surrounding biomaterial environment helps localize and support the regenerative signal.

These examples suggest that the value of hybrid systems lies in assigning different functions to different material components rather than expecting a single material to perform every task. An inorganic phase may provide antimicrobial, antioxidant, or mineralizing activity, while a polymeric phase can help control the presentation and release of therapeutic signals. This may be particularly useful in regenerative endodontics, where infection control needs to be followed by inflammation resolution, vascular recovery, matrix reconstruction, and odontogenic differentiation rather than maximizing all these processes simultaneously. However, this added complexity can make it difficult to determine which component is responsible for a particular biological effect. Future studies should therefore compare polymer-only, inorganic-only, and hybrid formulations to establish whether hybridization provides a meaningful advantage over simpler carrier systems.

### 4.7 Stimuli-responsive nanoparticles as the next stage of functional evolution

The progression of the strategies discussed above points from controlled delivery toward microenvironment-responsive regulation. Pathological pulp tissue provides endogenous triggers, particularly acidic pH, excessive ROS/RONS, and bacterial or inflammatory proteases, that can be exploited to alter nanoparticle degradation, intracellular trafficking, or cargo release. This principle is already evident across regenerative systems: protease-responsive materials can couple antimicrobial release to infection-associated enzymatic activity (41, 42), redox-active nanocomposites can reduce excessive RONS while modulating the inflammatory and macrophage environment (46), and pH-sensitive polymeric vectors can exploit endosomal acidification to facilitate intracellular delivery of regenerative genetic cargo (52). Collectively, these approaches indicate a functional evolution from fixed-release carriers toward adaptive nanotherapeutic systems whose behavior is determined by the biological state of the pulp.

For regenerative endodontics, the next step is not simply greater responsiveness, but temporal coordination of regenerative functions. An ideal polymeric platform could suppress antimicrobial and anti-inflammatory activity as infection resolves, enhance angiogenic support during vascular insufficiency, and subsequently favor matrix reconstruction and odontogenic differentiation. Such sequential regulation has not yet been demonstrated within a single endodontic nanoparticle system and therefore remains a design objective rather than an established capability. Nevertheless, it provides a framework for moving polymeric nanoparticles beyond therapeutic reservoirs toward microenvironment-responsive regulators of the regenerative sequence. The functional evolution of polymeric nanoparticle systems from passive drug carriers toward multifunctional, stimuli-responsive, and adaptive hybrid platforms is summarized in Figure 4.

**Figure 4:**
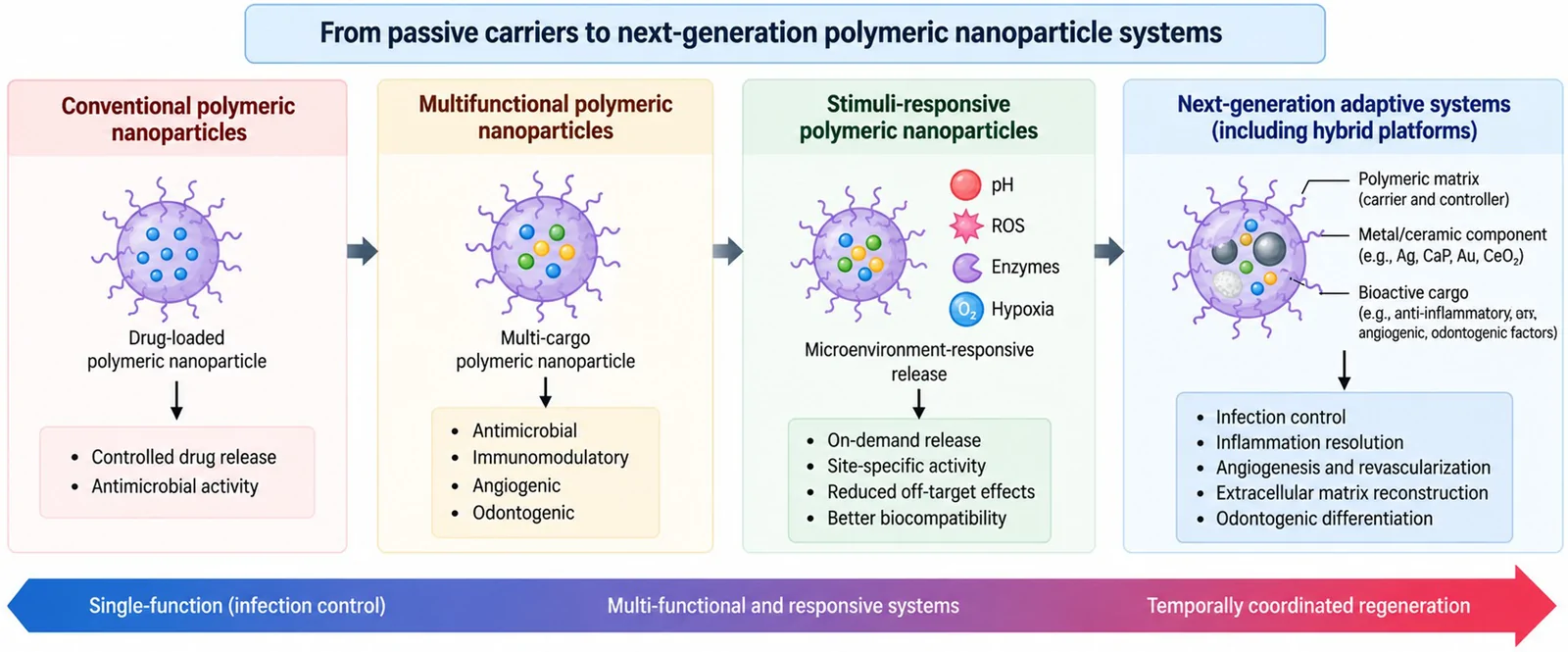
Functional evolution of polymeric nanoparticle systems for regenerative endodontics. Polymeric nanoparticles have progressed from conventional single-cargo carriers focused primarily on infection control toward multifunctional and stimuli-responsive systems capable of modulating several components of the pulp microenvironment. Next-generation adaptive and hybrid platforms may combine polymeric matrices with metal, ceramic, or other bioactive components to coordinate infection control, inflammation resolution, angiogenesis, extracellular-matrix reconstruction, and odontogenic differentiation. [Figure assembled using BioRender.].

## 5 Discussion: beyond antimicrobial therapy toward integrated microenvironmental control

The evidence synthesized above indicates that regenerative endodontic outcomes depend on coordinated control of cell recruitment, inflammation, vascularization, matrix reconstruction, and odontogenic differentiation rather than antimicrobial efficacy alone (61). Experimental nanoparticle studies further show that temporal presentation of bioactive molecules can alter stem-cell differentiation (34), while combined inflammation-modulating and regenerative signals can improve cellular responses under inflammatory conditions (62). The discussion therefore turns to whether polymeric systems can coordinate these functions in space and time, and to the translation barriers that currently separate this concept from clinical application.

### 5.1 From multifunctional delivery to coordinated microenvironmental regulation

The central challenge is therefore no longer simply whether a nanocarrier can perform more than one therapeutic function, but whether those functions are delivered in a biologically appropriate sequence. Pulp regeneration requires different signals at different stages: infection and excessive inflammation must first be controlled without eliminating the cells required for repair; vascular and trophic support must then permit tissue survival and ingrowth; and later signals must favor matrix reconstruction and odontoblast-lineage differentiation. Growth-factor biology already supports this temporal view because individual factors regulate distinct processes such as cell recruitment, angiogenesis, proliferation, and differentiation. Their spatial and temporal expression during cell homing is unlikely to be interchangeable (61). Thus, combining several cargos within one material does not by itself reproduce the regenerative microenvironment; their availability, duration, and biological context are equally important.

Direct nanoparticle evidence illustrates why timing matters. Chitosan nanoparticles loaded with dexamethasone generated different release profiles depending on whether the drug was encapsulated or surface-adsorbed, with both formulations sustaining release for four weeks but producing different temporal patterns of SCAP biomineralization and odontogenic-marker expression (34). This demonstrates that altering release kinetics without changing the cargo can change the cellular outcome, supporting temporal presentation itself as an engineering variable rather than a secondary formulation characteristic.

A complementary multifunctional system combined miR-146a-loaded PEG–PEI nanoparticles with bFGF in an alginate hydrogel under LPS-induced inflammatory conditions. The combined formulation increased DPC proliferation and odontogenic differentiation more effectively than either component alone, including increased DMP-1/DSP expression and mineral deposition (62). However, more than 70% of the incorporated cargo was released within the first 12 h before slower release continued. This means that the system demonstrated co-delivery of complementary inflammatory and regenerative cues rather than true stage-dependent sequential regulation (62).

This distinction is important for the next generation of polymeric nanoparticles. Current smart dental biomaterials already show that environmental responsiveness can provide localized or on-demand release, while multifunctional scaffolds can combine antimicrobial, immunomodulatory, angiogenic, and regenerative functions (63). More broadly, stimuli-responsive carriers are being designed to alter release according to changes in the local microenvironment and thereby provide greater spatiotemporal control than conventional sustained-release systems (63).

Taken together, these findings support a proposed shift from simultaneous multifunctionality toward coordinated multifunctionality. In this model, antimicrobial or inflammation-dampening activity would predominate while pathological signals persist, followed by increasing angiogenic, matrix-supportive, and fate-directing activity as the tissue moves toward repair. No study identified in this review demonstrates this complete sequence within a single endodontic polymeric nanoparticle platform. This coordinated model therefore represents a design framework emerging from the combined evidence rather than an established therapeutic capability. The ultimate objective is a system in which therapeutic function is matched not simply to the presence of a cargo, but to the changing biological state of the pulp.

### 5.2 Translational barriers and clinical considerations

Despite increasingly sophisticated nanoparticle designs, a major barrier to clinical translation is the gap between controlled experimental performance and the biological complexity of the human root canal. In a recent scoping review of endodontic nanoparticles, 75% of included studies were performed *in vitro* and only 25% incorporated animal models, with most investigations centered on odontogenic differentiation, material enhancement, or antimicrobial activity rather than functional pulp regeneration (64). Moreover, laboratory models often fail to reproduce the anatomical, microbial, vascular, and mechanical conditions of the dentin–pulp complex. More representative whole-tooth, multispecies-biofilm, and orthotopic animal models are therefore needed before regenerative efficacy can reasonably be extrapolated to patients (64). This limitation is especially relevant to polymeric nanoparticles intended to regulate the pulp microenvironment, because therapeutic performance will depend not only on cargo activity but also on penetration, retention, degradation, and interaction with host cells under clinically relevant inflammatory and microbial conditions.

These challenges are amplified by the anatomy of the root canal itself. Therapeutic materials must function within a confined and anatomically variable space in which access to the regenerating tissue is restricted and vascular supply is largely dependent on the apical region. For nanoparticle-based systems, this means that biological activity demonstrated under simplified culture conditions may not translate directly into adequate distribution, retention, or interaction with target cells within a full-length root canal. More representative whole-tooth and orthotopic models are therefore particularly important for determining whether nanoparticle formulations can maintain their intended function under clinically realistic anatomical and microbial conditions (64).

A similar translational gap is evident in extracellular-vesicle-based oral regeneration. Despite promising effects on immune regulation, angiogenesis, and tissue repair, the dental stem-cell-derived EV literature remains dominated by *in vitro* studies and small-animal proof-of-concept models, with scarce large-animal evidence and no completed Phase II or III clinical trials for DMSC-EVs in oral tissues (65). This parallel is relevant to nanoparticle-based regenerative endodontics because it illustrates that strong biological activity in simplified models does not itself establish clinical translatability (65).

Safety must likewise be evaluated beyond short-term cytocompatibility. Polymeric carriers can reduce local drug exposure and improve therapeutic efficiency, but particle concentration, degradation kinetics, cargo release, and the biological effects of degradation products must remain compatible with surviving pulp, apical, vascular, and immune cells. A useful endodontic example is berberine-loaded PLGA nanoparticles, which achieved antimicrobial effects at substantially lower berberine concentrations, showed negligible toxicity toward gingival fibroblasts, and retained physicochemical stability and approximately 95% of the incorporated drug during 20 weeks of storage at both 4 °C and room temperature (66). These findings demonstrate that stability and cytocompatibility can be engineered into a polymeric formulation, but they remain preclinical formulation-level evidence rather than proof of long-term intrapulpal safety. Indeed, the broader endodontic evidence base continues to identify long-term biocompatibility, scalability, and integration into clinical workflows as unresolved barriers (64).

Translation also requires a shift from laboratory-scale formulation to a reproducible therapeutic product. Increasing architectural and functional complexity may improve biological performance while simultaneously making manufacturing more difficult. Particle size distribution, surface properties, cargo loading, release kinetics, degradation behavior, and batch-to-batch consistency must remain reproducible after scale-up. Across regenerative biomaterials, complex fabrication and insufficient standardization are associated with high production costs and limited reproducibility, while regulatory translation is hindered by non-harmonized guidelines and prolonged approval pathways (67). These concerns are mirrored in the emerging DMSC-EV field, where differences in isolation procedures produce substantial variation in purity, yield, and biological activity. Donor- and passage-dependent cargo heterogeneity and the absence of a unified dosing metric further complicate product standardization (65). Together, these observations reinforce that biological sophistication must be accompanied by clearly defined and reproducible quality attributes if regenerative nanotherapies are to progress toward clinical use (65). For multifunctional polymeric nanoparticles, this creates an important design trade-off: every additional responsive component, targeting ligand, or therapeutic cargo may increase biological capability but also increase the number of critical quality attributes that must be controlled. Consequently, translationally advanced does not necessarily mean maximally complex; clinically credible systems will need to balance microenvironmental responsiveness with manufacturability, storage stability, quality control, and practical chairside use.

A further gap lies between regenerative efficacy in experimental models and evidence for clinically meaningful pulp restoration. Ectopic and semiorthotopic models are useful mechanistically but differ substantially from clinical conditions in blood supply, operative environment, and cellular contribution, whereas orthotopic large-animal models more closely reproduce the human root canal but introduce greater cost, technical difficulty, and ethical constraints (68). Clinical pulp-regeneration studies remain limited and often involve cell-based strategies requiring specialized processing or GMP facilities. Even promising autologous approaches have been constrained by cost and logistical complexity and still require larger clinical trials (68). For nanoparticles specifically, clinical evidence is even less mature: only two randomized endodontic trials identified in the 2025 scoping review evaluated nanoparticle-based antibacterial outcomes, while regenerative nanoparticle applications remained essentially preclinical (64).

Accordingly, the translational benchmark for next-generation polymeric nanoparticles should extend beyond demonstrating antimicrobial activity, increased differentiation markers, or favorable short-term cytocompatibility. A clinically relevant platform should show reproducible manufacture and storage, predictable degradation and release, safety across relevant pulp-cell populations, activity in multispecies and inflammatory environments, retention within the root canal, and ultimately regeneration in orthotopic models using endpoints that distinguish vascularized, innervated pulp–dentin tissue from nonspecific mineralized repair. The central translational challenge is therefore not simply to make polymeric nanoparticles more multifunctional, but to determine the minimum controllable complexity required to achieve reproducible biological regulation under clinically realistic conditions.

This review also has limitations. As a narrative review, study selection was not based on a formal systematic-review protocol and may therefore be subject to selection bias. Direct evidence for polymeric nanoparticles in regenerative endodontics also remains limited, requiring consideration of mechanistically relevant non-polymeric, non-endodontic, and preclinical studies. The heterogeneity of these materials, models, and outcome measures limits direct comparison between studies, and some of the proposed design principles should therefore be regarded as future directions rather than established endodontic strategies.

### 5.3 Future perspectives: toward adaptive pulp-microenvironment engineering

The next step in regenerative endodontic nanotechnology is likely to be not simply greater multifunctionality, but closer coupling between therapeutic activity and the changing biological state of the pulp. Microenvironment-responsive nanosystems can already exploit pathological cues such as acidic pH, altered redox conditions, and increased enzymatic activity to control local cargo release, providing a basis for moving from predetermined delivery toward condition-dependent regulation (69). Such adaptive regulation may be particularly important because inflammatory signaling in DPSCs is not uniformly detrimental. Recent analysis of the inflammation–regeneration axis indicates that Wnt/β-catenin and NF-κB signaling interact in a context-dependent manner. Low-dose LPS (<1 μg/mL) can support reparative responses through coordinated pathway activation, whereas sustained higher-dose exposure (>1 μg/mL) can suppress Wnt signaling through NF-κB-driven DKK1 upregulation and attenuate DPSC differentiation (30). Rather than constitutively suppressing inflammatory pathways, future nanosystems may therefore need to distinguish between signaling compatible with repair and persistent inflammatory states that inhibit regeneration (30).

Regeneration is also temporally organized, and evidence from tissue-engineering models shows that nanoparticle–hydrogel systems can separate an early immunomodulatory phase from a later regenerative phase. Sequential delivery of IL-10 followed by sustained icariin release, for example, promoted immune regulation before osteogenic differentiation and tissue repair (70). This concept is also consistent with reciprocal DPSC–immune-cell signaling: DPSC-derived Wnt3a has been associated with macrophage polarization toward an M2 phenotype, whereas M2-derived Wnt7b can enhance DPSC odontogenic differentiation while suppressing NF-κB expression (30). Such bidirectional signaling suggests that future nanocarriers could be designed not merely to deliver a fixed anti-inflammatory or odontogenic signal, but to coordinate the transition from inflammatory resolution to tissue regeneration (30). By analogy, a future pulp-directed system could allow antimicrobial and inflammation-resolving functions to dominate while pathological signals persist, followed by increasing angiogenic, matrix-supportive, and odontogenic activity as tissue conditions improve.

This level of sequential adaptation has not yet been demonstrated in a single regenerative-endodontic polymeric nanoparticle system and therefore remains a design objective rather than an established capability.

Computational design may provide an additional route toward greater precision. Machine-learning approaches have already been combined with experimental design to identify formulation variables governing PLGA nanoparticle properties and improve prediction of particle size (71). More broadly, AI in dentistry is increasingly moving toward integration of heterogeneous clinical and biological data for individualized prediction and treatment planning, although external and prospective validation remains limited (72). In the longer term, coupling such patient-specific information with nanoparticle formulation models could help match carrier composition, release kinetics, and therapeutic sequence to differences in microbial burden, inflammatory status, and regenerative capacity. This remains prospective, but it defines a useful direction for the field: from smart delivery toward adaptive regulation of the pulp microenvironment.

## 6 Conclusion

Polymeric nanoparticles have the potential to move regenerative endodontics beyond disinfection by influencing the biological conditions that determine whether damaged pulp undergoes repair or true regeneration. However, the current evidence remains predominantly preclinical, and direct evidence for polymeric nanoparticles in regenerative endodontic models is still limited. Many of the multifunctional and microenvironment-responsive principles discussed in this review are supported by *in vitro* studies, animal models, or mechanistically relevant non-polymeric and non-endodontic systems and should therefore be regarded as promising design principles rather than established therapeutic capabilities.

The available evidence nevertheless indicates that infection control, immune regulation, vascular recovery, extracellular-matrix reconstruction, and stem-cell fate are interconnected processes whose timing and interaction are likely to influence regenerative outcomes. Current systems have individually demonstrated controlled release, microenvironment responsiveness, immunomodulation, angiogenic support, and regulation of odontogenic differentiation, but no single polymeric nanoparticle platform has yet coordinated these functions across the full regenerative sequence. Near-term research should therefore prioritize direct evaluation of polymeric nanoparticles in regenerative endodontic models, validation in whole-tooth and orthotopic models, assessment of penetration, retention, degradation, and release under clinically relevant root-canal conditions, and evaluation of long-term safety, reproducibility, and compatibility with clinical workflows.

The goal should not be to increase material complexity for its own sake, but to establish reproducible systems that provide the appropriate biological function at the appropriate stage of healing. Once these fundamental requirements are demonstrated, more sophisticated sequential or microenvironment-responsive platforms may provide a route toward adaptive regulation of the pulp microenvironment.
